# Tag7 (PGLYRP1) Can Induce an Emergence of the CD3+CD4+CD25+CD127+ Cells with Antitumor Activity

**DOI:** 10.1155/2018/4501273

**Published:** 2018-04-11

**Authors:** T. N. Sharapova, E. A. Romanova, L. P. Sashchenko, D. V. Yashin

**Affiliations:** Institute of Gene Biology, RAS, 34/5 Vavilova Str, Moscow 119334, Russia

## Abstract

We have shown that in the human peripheral blood cells, the innate immunity protein Tag7 can activate a subpopulation of CD3+CD4+CD25+ cells, which have antitumor activity. These cells can induce lysis of HLA-negative tumor cell lines. The Hsp70 stress molecule on the surface of the tumor cells is used as a recognition target, while the Tag7 protein on the lymphocyte membrane acts as a receptor for Hsp70. We have also demonstrated that this subpopulation of the CD4+CD25+ cells is CD127 positive and hence is not the Treg cells. Our data suggest that this subpopulation of cells is identical to the CD4+CD25+ lymphocytes, which are activated in the leukocyte pool by the IL-2 cytokine.

## 1. Introduction

It is now clear that the capabilities of the classical T lymphocytes (CTL) are inadequate for their use in anticancer therapy. Classical CD8+ T cells specifically detect pathogens and tumor peptide antigens presented via the MHC (HLA) class I molecule; however, tumor cells often use a strategy known as immune evasion [1]. They can block, due to mutations, the cell death transduction pathways or change the repertoire of antigens on the cell surface. In the most radical case of evasion, they completely lose their HLA components and become completely unrecognizable to the CTL [2].

To contend with these phenomena, the body has several defense mechanisms. In addition to the classical CTL, several specialized subpopulations of lymphocytes were described that can recognize and kill the HLA-negative cells. These include the NK cells of the innate immune system [3]. Besides, there are cells, which are at the boarder of the innate and adaptive immunity, the NKT cells and *γδ* lymphocytes [4–6]. However, these protective systems are not perfect, and a search for lymphocytes able to deal with the immune evasion is essential not only for a comprehensive understanding of the immune defense mechanisms but also for the identification of new immunotherapeutic agents. Attention should be paid to specific subpopulations of lymphocytes. It is known that the CD8+ T lymphocytes, which have the NK-activating receptor NKG2D on their surface, acquire an NK-like activity and the ability to kill the HLA-negative tumor cells after a prolonged incubation with the IL-15 or IL-2 cytokines [7–9].

According to our data, a prolonged incubation of lymphocytes with IL-2 leads to an activation of a subpopulation of CD4+CD25+ cells, which is able to kill HLA-negative tumor cells through the FasL-Fas interaction [10]. For a long time, the CD4+ T lymphocytes have been considered the only regulatory cells, due to their ability to secrete cytokines that regulate various processes of the immune response. Cytokines can promote phagocytic activity, generate cytotoxic CD8+ T cells, regulate the production of antibodies and inflammation, and can also suppress the immune response. Recently, it has been clearly demonstrated that CD4+ T cells have cytotoxic activity. Under certain chronic inflammatory conditions, increasing numbers of CD4+ T cells may undergo progressive differentiation, during which cells lose expression of the costimulatory molecules CD28 and CD27 and gain expression of intracellular cytotoxic granules and perforin [11, 12]. These CD28−granzyme+perforin+CD4+ T cells recognize antigenic peptide in the context of MHCII and kill antigen-carrying cells via perforin-granzyme secretion [13]. Regulatory CD4+CD25+ T cells, whose function is to suppress effector T lymphocytes, constitute a special Treg group [14]. The mechanism of the Treg suppressor effect is not completely clear. It is known that the regulatory CD4+CD25+ subpopulations usually secrete suppressor cytokines that inhibit the activity effector cells. However, it has been suggested that Treg lymphocytes can have a direct cytotoxic effect on the cells, releasing perforin and granzymes [15]. However, no connection has been established between the cytotoxic CD4+CD25+ lymphocytes that we have described [10] and the Treg cells.

To understand the defense mechanisms employed by an organism to guard itself from tumor cells that escape immune control, it is important to consider regulatory protein molecules secreted by cells of the immune system. The innate immunity protein Tag7, also known as peptidoglycan-recognition protein or PGRP-S (PGLYRP1), is involved in the antimicrobial and antitumor protection systems [16, 17]. The gene coding for this protein was identified for the first time in our laboratory [18]. It is found in insects and mammals, and its structure is conserved [19]. Its role in the mechanism of antibacterial protection is well studied in insects. It was shown that Tag7 interacts with the Toll-like receptor, followed by activation of the transcription factor NF-*κ*B and subsequent production of antibacterial peptides [20].

We have studied the Tag7 participation in the antitumor protection and found that this protein demonstrates many new functional activities. We have shown that in a complex with the major heat shock protein Hsp70, it has a direct cytotoxic effect on some tumor cell lines [21]. The Tag7-Hsp70 complex is secreted by cytotoxic lymphocytes and inhibits tumor growth [21]. In a complex with the Ca^2+^-binding protein Mts1 (S100A4), Tag7 has chemoattractant activity [22]. On the surface of the CD4+CD25+ lymphocytes, Tag7 acts as a specialized receptor involved in the recognition of HLA-negative tumor cells. Recently, we have shown that it is able to induce cytotoxic activity of lymphocytes [23].

The aim of this study was to determine whether Tag7 can activate a subpopulation of the CD4+CD25+ cytotoxic cells, to describe possible mechanisms of the cytotoxic action of these cells, and to determine whether the Treg fraction of CD4+CD25+ cells has cytotoxic activity.

## 2. Materials and Methods

### 2.1. Cell Culture and Sorting

K562 cells (human erythroblastoma), MOLT-4 cells (human lymphoblastoma), HeLa cells, and the L929 cells (murine fibroblasts) were cultured in DMEM and RPMI-1640, with 2 mM L-glutamine and 10% fetal calf serum (all from Invitrogen, Carlsbad, CA). Human PBMCs (peripheral blood mononuclear cells) were isolated from the total healthy donor's leukocyte pool by the Ficoll-Hypaque density gradient centrifugation, as described [24], and cultured in RPMI-1640 at a density of 4 × 10^6^ cells/mL (see above) either with the recombinant IL-2 (1000 U/mL) or with Tag7 (10^−9^ M) for 6 days. Cell sorting was performed using standard magnetic bead kits (Dynal Biotech ASA, Oslo, Norway) according to the manufacturer's protocol. Results of cell sorting were always verified by flow cytometry, and there were no experiments with less than 80% purity. Specific antibodies to CD127 or Tag7 were coupled to magnetic beads coated with the anti-rabbit antibodies, following the methods of Sashchenko et al. [25].

### 2.2. Proteins and Antibodies

The human IL-2 cDNA was subcloned in pQE-30 plasmid and expressed in *E. coli* M15 cells. Polyclonal antibodies to the N-terminus of Fas (N-18) and C-terminus of FasL (С-178) were from Sigma (United States); CD4-PE, CD4-FITC, CD4-TC, CD25-FITC, CD127-PE, FasL-FITC, FasL-PE, and Fas-PE were from Caltag Medsystems (Buckingham, UK). Control antibodies were IgG from rabbit serum (Sigma-Aldrich, St. Louis, MO), following the methods of Sashchenko et al. [25].

### 2.3. Cytotoxicity Assays

The K562 cells were cultured in the 96-well plates (3 × 10^4^ cells per well) and mixed with lymphocytes added at a 20 : 1 (20 lymphocytes to 1 target cell) ratio and incubated at 37°C for 20 h. The effector-to-target ratio was selected based on our previous experiments (see Supplemental Figure 1). The inhibition test was conducted with the polyclonal antibodies (anti-Tag7, anti-Hsp70, anti-Fas, anti-FasL, and anti-granzyme B) at a concentration of 20 *μ*g/mL. Cytotoxicity was measured with a Cytotox 96 Assay kit according to the manufacturer's instructions (Promega, Madison, WI), always subtracting the percentage of dead cells (lymphocytes or target cancer cells) (within 5%).

### 2.4. Flow Cytometry

**Figure 1:**
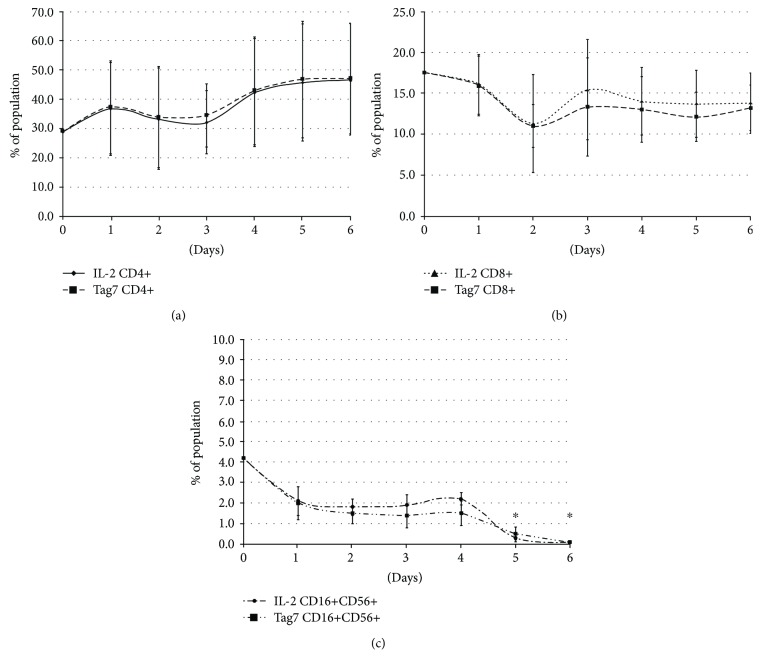
Flow cytometry of the PBMC pool on days 1 to 6 of its incubation either with IL-2 (1000 units/mL) or with the Tag7 protein (10^−9^М), stained with (a) CD3-FITC and CD4-PE, (b) CD3-FITC and CD8-PE, and (c) CD56-FITC and CD16-PE. The percentage of the double-positive cells is shown. Statistics: two-way ANOVA versus control (^∗^ *P* < 0.05, *n* = 5).

**Figure 2 fig2:**
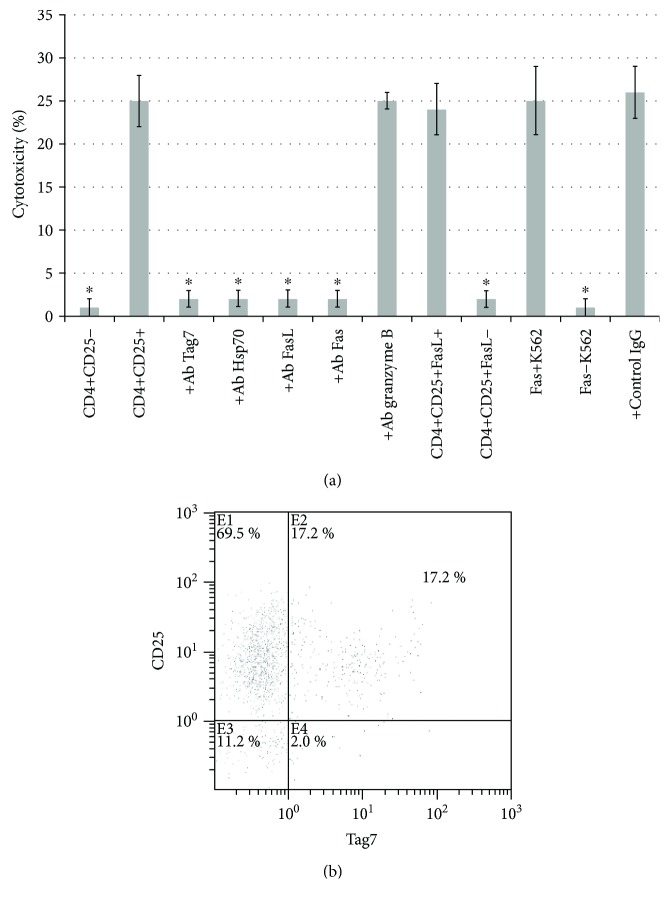
Tag7 participates in cytotoxic activity of CD4+CD25+ cells. (a) The cytotoxic activity of the CD4+ lymphocyte subpopulations (CD4+CD25− and CD4+CD25+) against the K562 cells and the cytotoxic activity of the CD4+CD25+ subpopulation after a 30 min preincubation of the lymphocytes with anti-granzyme B, FasL, and anti-Tag7 antibodies, or the control Ab, and after a 30 min preincubation of the K562 cells with anti-Fas or anti-Hsp70 antibodies. Statistics: two-way ANOVA versus control (^∗^ *P* < 0.05, *n* = 5). Control cell death was 1 ± 1 for lymphocytes and 3 ± 1 for K562 cells. (b) Flow cytometry of the CD4+CD25+ T lymphocytes, isolated from the PBMC pool on day 6 of incubation with Tag7 and stained with the rabbit anti-Tag7 antibodies followed by the FITC-conjugated anti-rabbit antibodies and CD25-PE.

**Figure 3:**
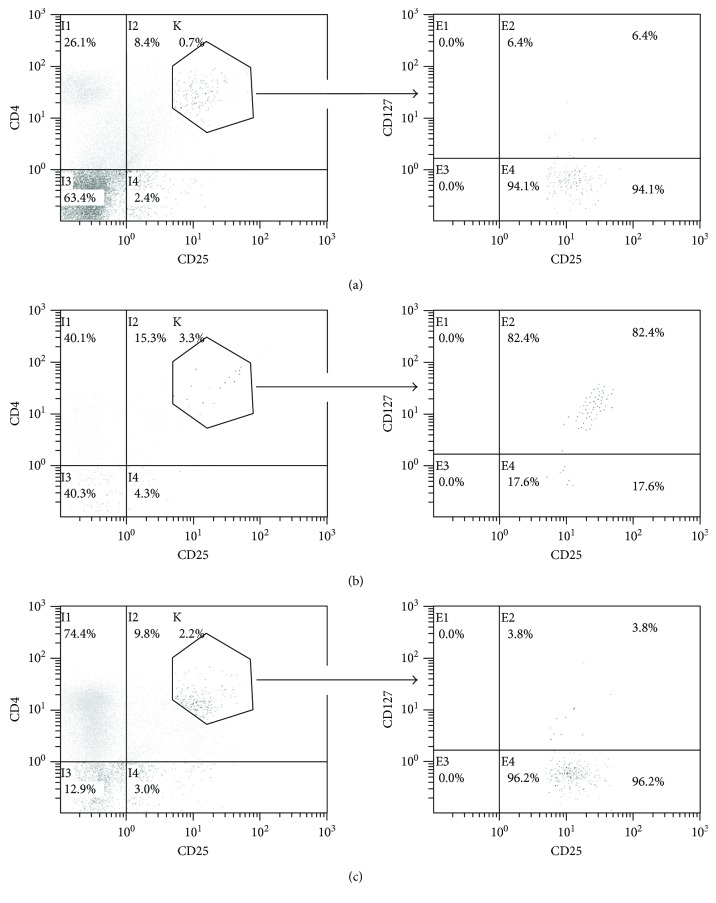
Flow cytometry of the total PBMC pool on day 6 of incubation with Tag7 (a), the Tag7-positive subpopulation isolated via magnetic separation (b), and the Tag7-negative subpopulation (c) stained with the CD25-FITC, CD127-PE, and CD4-TC antibodies. The left panel shows the staining with the anti-CD25 and anti-CD4 antibodies, and the right panel shows the staining of the CD4+CD25^high^ subpopulation from the left panel with the anti-CD127 and anti-CD25 antibodies.

The cells were routinely fixed with 1% paraformaldehyde (Sigma-Aldrich, St. Louis, MO) and stained with antibodies at 18°C. To test for intracellular FoxP3, lymphocytes were fixed and permeabilized using the Human Regulatory T Cell Staining Kit (eBioscience) following the manufacturer's instructions. The cells (at least 10^4^ cells per test) were analyzed with an Epics Elite flow cytometer (Coulter, Marseille, France) in the logarithmic channel of fluorescence. The data were processed with EXPO32 software (Applied Cytometry Systems, Sheffield, UK). The gating strategy and controls for Figures 1 [Fig fig2]–3 are described in Supplemental Figures 2–4.

### 2.5. Statistical Analysis

Data are presented as the average ± standard deviation. All data were calculated from at least five independent biological replicates. Testing for significant differences between treatment and control was performed with MathCad software (PTC, Cambridge, MA). The statistical tests were carried out as stated in the legends of the corresponding table or figure, employing the two-way ANOVA test.

## 3. Results

### 3.1. The CD3+CD4+CD25+ Fraction of Cells Is Responsible for the Tag7-Activated Cytotoxicity of the CD3+CD4+ Lymphocytes

In order to figure out if Tag7 can activate lymphocytes, we performed a comparative analysis of the accumulation of CD3+CD4+ cells in the subpopulation of human PBMC, induced either by the IL-2 cytokine or by the Tag7 innate immunity protein. PBMCs were incubated with either IL-2 or Tag7 for 6 days, and every 24 h, the percentage of standard markers, CD3 and CD4, was determined on the cell surface by flow cytometry.

As seen from the results shown in Figure 1(a), the CD3+CD4+ lymphocytes represent a significant portion of the PBMC, and their proportion increases with incubation time, reaching a maximum on the sixth day. At the same time, the general shape of the curve and the obtained values for the CD3+CD4+ numbers of cells are not that much different for the activation by either the IL-2 cytokine or the Tag7 protein. These data claim that both the cytokine of adaptive immune response IL-2 and the innate immunity protein Tag7 induce in PBMC similar proportion of CD3+CD4+ cells.

For comparison, we tested the expression of surface markers CD3, CD8, CD16, and CD56 after incubation with each inducer (Figures 1(b) and 1(c)). It can be seen that the curve profiles are identical for induction with IL-2 and Tag7 but differ for different cell subpopulations. The proportion of CD3+CD8+ lymphocytes does not change for 6 days of incubation. The proportion of NK cells is substantially lower but becomes slightly increased on the 4th day of incubation. On the 6th day of incubation, NK cells disappear. Thus, the studied population on the 6th day contains CD3+CD4+ lymphocytes and CD3+CD8+ lymphocytes and does not contain NK cells. Thus, 6-day PBMC cytotoxicity depends only on CD3+CD4+ and CD3+CD8+ lymphocytes.

**Figure 4:**
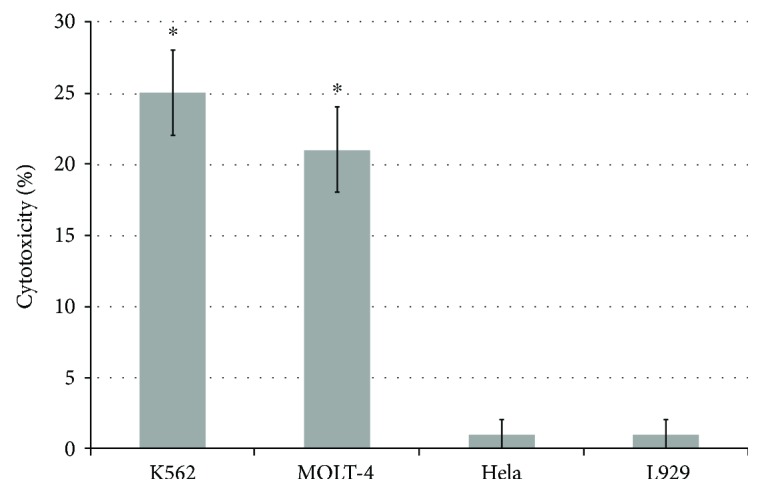
The cytotoxic activity of the CD4+ T lymphocytes against the K562, MOLT-4, L929, and HeLa cells. Statistics: one-way ANOVA versus control (^∗^ *P* < 0.05, *n* = 5). Control death of cells was 3 ± 1 for K562, 5 ± 2 for MOLT-4, 2 ± 1 for HeLa, 2 ± 1 for L929, and 1 ± 1 for lymphocytes.

Next, we questioned whether the Tag7-activated CD3+CD4+ lymphocytes are able to kill tumor cells that had escaped the immune control. Using magnetic separation, we have isolated the CD3+CD4+ cell fraction from the total PBMC pool after 6 days of incubation with the Tag7 protein. The isolated lymphocyte fraction was added to various tumor cell lines and was tested for cytotoxic activity. The following cell lines were used: K562, MOLT-4, HeLa, and L-929. The Tag7 protein-activated CD3+CD4+ lymphocytes only killed the HLA-antigen-lacking K562 and MOLT-4 cells (Figure 4). At the same time, we have not found any trace of cytotoxic activity against the HeLa and L929 cells, carrying HLA antigen, or their mouse analogue. The highest cytotoxic activity was observed against the K562 cells (about 25% of dead cells). Thus, the Tag7-activated CD3+CD4+ subpopulation of lymphocytes was only able to kill HLA-negative tumor cells, as has also been demonstrated for the cytokine IL-2-activated lymphocytes [10].

Previously, we have shown that on the sixth day of incubation with IL-2, the CD4+CD25+ subpopulation of lymphocytes was activated. Therefore, we wondered whether the Tag7-activated CD4 T lymphocytes are also CD25 positive. For this purpose, using magnetic separation, we have isolated subpopulations of the CD3+CD4+CD25+ and CD3+CD4+CD25− cells after 6 days of incubation of the PBMC with Tag7. The cytotoxic activity was demonstrated only by the CD3+CD4+CD25+ subpopulation, while the CD3+CD4+CD25− subpopulation was unable to cause lysis of the K562 cells (Figure 2(a)). Thus, these results indicate that both the IL-2 cytokine and the Tag7 innate immunity protein are able to induce cytotoxic activity of the CD3+CD4+CD25+ lymphocytes after six days of incubation.

### 3.2. The Cytotoxic Activity of the CD3+CD4+CD25+ Lymphocytes Requires the Presence of FasL and Tag7 on the Lymphocyte Surface and a Simultaneous Presence of Fas and Hsp70 on the Surface of the Target Cell

To confirm the identity of the CD3+CD4+CD25+ lymphocyte fractions activated by either the IL-2 or Tag7, a more detailed investigation of a mechanism of cytotoxic activity of these cells was carried out.

Previously, we have shown that CD3+CD4+CD25+ lymphocytes activated by IL-2 killed HLA-negative tumor cells through the FasL-Fas interaction [23]. Here, we have tested if this is true for the CD3+CD4+CD25+ subpopulation, activated by Tag7 protein. Indeed, the preincubation of the CD3+CD4+CD25+ T lymphocytes with the anti-FasL antibodies or the preincubation of the K562 cells with the anti-Fas antibodies resulted in a complete disappearance of the cytotoxic activity (Figure 2(a)). The removal of the FasL-bearing lymphocytes and Fas-expressing K562 cells by magnetic bead separation also resulted in the disappearance of the cytotoxic activity. The incubation of the cells with the anti-granzyme B antibodies had no effect on cytotoxicity. It should be noted that these antibodies could inhibit cytotoxicity of NK cells, purified from the PBMC on 4 days of incubation with Tag7 protein (Supplemental Figure 5). These results indicate that, similar to the IL-2-activated lymphocytes, the CD3+CD4+CD25+ lymphocytes kill their target cells through the FasL-Fas interaction.

Next, we determined how these cells recognize their targets. We have previously shown that the cytokine IL-2-activated CD3+CD4+CD25+ lymphocytes carry the innate immunity protein Tag7, which recognizes the Hsp70 heat shock protein on the surface of the K562 cells. We have previously demonstrated the presence of the Hsp70 protein on the surface of the K562 cells [10]. Here, using flow cytofluorimetry, we investigated whether Tag7 is present on the surface of the CD3+CD4+CD25+ T lymphocytes, which were activated by the Tag7 protein. As seen in Figure 2(b), about 17% of the CD3+CD4+CD25+ T cells also carry Tag7.

Next, we tested whether Tag7 of lymphocytes and Hsp70 on the surface of the K562 cells are involved in the cytotoxic process. It is evident (Figure 2(a)) that the preincubation of the CD3+CD4+CD25+ lymphocytes with the specific antibodies against Tag7 or the preincubation of the K562 cells with the anti-Hsp70 antibodies blocked the cytotoxic activity, suggesting that the binding of the lymphocytic Tag7 with the Hsp70 on the tumor cell surface is necessary for its subsequent killing through the FasL-Fas interaction. Addition of the control antibodies had no effect on the cytotoxicity of this lymphocyte population. As an additional control, we have shown that cytotoxicity of other T cell fraction—CD8+ T lymphocytes—is not blocked by anti-Tag7 or anti-Hsp70 antibodies (Supplemental Figure 6).

### 3.3. The Tag7-Activated CD3+CD4+CD25+ Cytotoxic Lymphocytes Are Not Treg Cells

**Figure 5:**
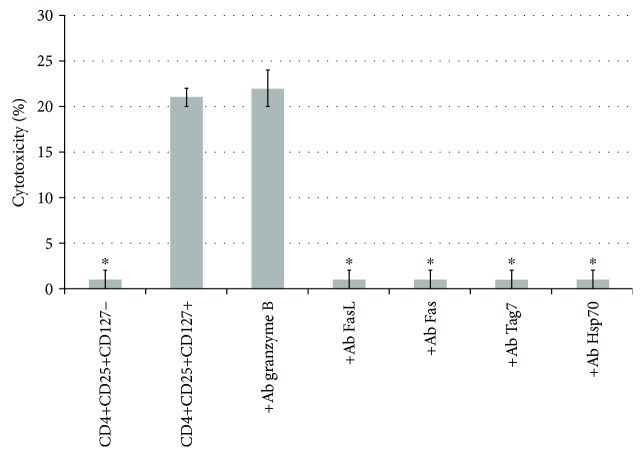
The cytotoxic activity of the CD4+CD25+ lymphocyte subpopulations (CD4+CD25+CD127− and CD4+CD25+CD127+) against the K562 cells and the cytotoxic activity of the CD4+CD25+CD127+ subpopulation after a 30 min preincubation of the lymphocytes with anti-FasL antibodies or anti-granzyme B antibodies or after a 30 min preincubation of the K562 cells with the anti-Fas antibodies. Statistics: two-way ANOVA versus control (^∗^ *P* < 0.05, *n* = 5). Control cell death was 1 ± 1 for lymphocytes and 3 ± 1 for K562 cells.

It is known that the CD4+CD25+ lymphocytes are often associated with the Treg subpopulation of cells, which are responsible for the suppression of the immune response in the organism that enables tumor cell survival [26]. It has been shown that unlike other nonregulatory CD4+ cell populations, Treg cells in addition to the CD4 and CD25 antigens do not carry the CD127 antigen [27]. We decided to determine whether the cells that exhibit the antitumor activity in our experiments are Treg cells or not. To this end, we have isolated subpopulations of the CD3+CD4+CD25+, CD3+CD4+CD25+Tag7+, and CD3+CD4+CD25+Tag7− cells by magnetic separation and stained cells of the CD4+CD25^high^ phenotype with antibodies to CD127 (Figures 3(b) and 3(c)). As a control, the total Tag7-activated PBMC population was also stained (Figure 3(a)). As can be seen from the results of the cytofluorimetric analysis, the CD127 antigen was located on the same cells that carry Tag7 on their surfaces. To additionally verify this finding, we have stained the CD4+CD25+CD127+ subpopulation isolated with magnetic beads, for intracellular FoxP3 (Supplemental Figure 7). No significant increase of FoxP3 inside the cells was detected. Next, we have isolated subpopulations of the CD4+CD25+CD127+ and CD4+CD25+CD127− lymphocytes and tested their cytotoxic activity (Figure 5). The CD4+CD25+CD127+ subpopulation was active, causing death of 20% of the tumor cells, while the CD4+CD25+CD127− subpopulation did not demonstrate any cytotoxic properties. Antibodies to FasL, Fas, Hsp70, and Tag7 inhibit the cytotoxic activity of the CD4+CD25+CD127+ subpopulation, while antibodies to granzyme B have no effect, although we have detected granzymes inside of lymphocytes via intracellular antibody staining (Supplemental Figure 8). It seems that granzymes are not secreted during the interaction of this cytotoxic population with HLA-negative tumor cells.

Thus, we have demonstrated that in the human PBMC, after 6 days of incubation with the Tag7 protein, it can induce development of a subpopulation of the CD3+CD4+CD25+CD127−FasL+Tag7+ lymphocytes, which are not Treg cells. These cells have antitumor activity.

## 4. Discussion

We have recently demonstrated a new function of Tag7 in the immune response, that is, its ability to activate the cytotoxic lymphocytes in the human peripheral blood [23].

In this work, we have demonstrated that Tag7, after 6 days of incubation with PBMC, activates the CD4+CD25+ population of the cells different from the known CD4+ cytotoxic lymphocytes both by functional activity in immune defense and by mechanisms of cytotoxic action.

For a long time, it was believed that the regulatory function of the CD4+ T helpers is associated with either activation or inhibition of the effector lymphocytes. The heterogeneity of the CD4+ T lymphocyte population suggests a diversity of their functional activities. Previously, it was reported that the CTL CD4+ lymphocytes were detected only in T cell lines and clones in mice and humans, but those results have been deemed as artefactual [28, 29]. Recently, the cytotoxic CD4+ T lymphocytes were identified in the human PBMC, mostly during chronic viral infections [30–32]. These data show that one of the most important functions of CD4+ CTLs is an antiviral immune response, but they are also able to participate in antitumor immune response [33], chronic inflammatory, and autoimmune responses [34, 35].

Typically, they kill cancer cells that express MHCII on their surface [36]. We described a subpopulation of the CD4+CD25+ cytotoxic cells which is able to kill HLA-negative tumor cells; that is, these lymphocytes are involved in the fight against tumor cells that have escaped immune control.

The binding of classical cytotoxic T lymphocytes to their target cell is ensured by the interaction of TCR on the lymphocyte surface with the antigen in the context of MHC class I or II on the surface of the target cell. Cells that have lost MHC express stress proteins on their surface, which contribute to the recognition and subsequent killing of these cells by subpopulations of noncanonical lymphocytes. Stress proteins include noncanonical MHC protein families, Mic and ULP [37, 38], as well as the major heat shock protein Hsp70.

In healthy cells, it is present only in the cytoplasm. Currently, it is well known that Hsp70 appears on the surface of many HLA-negative tumor cells [39, 40] and it is bound to the lipid rafts on the cellular membrane through the protein-lipid interactions [41].

As was mentioned in Introduction, cytotoxic CD4+ cells recognize antigens on the surface of target cells in the context of MHCII [36]. We have demonstrated that Tag7-activated CD4+CD25+ lymphocytes kill the HLA-negative cells carrying Hsp70 protein on their surface. Tag7 on the surface of these lymphocytes interacts with Hsp70 on the surface of the target cells, ensuring the binding of these cells.

The mechanism of killing of target cells largely depends on the identity of proteins that are involved in the binding of these cells to lymphocytes. It is known that the interaction of MHC with TCR leads to a sharp increase in the intracellular concentration of Ca^2+^ ions, which ensures the release of granzyme-containing granules into the contact zone [42]. During the interaction of Tag7 with Hsp70, apparently, there is no increase in the concentration of Ca^2+^ ions inside the lymphocyte and there is no granzyme release, even if they are present there. These lymphocytes have FasL on their surface, and they kill HLA-negative target cells via the FasL-Fas interaction.

We first described the appearance of such CD4+CD25+ lymphocytes upon the activation of human PBMC by IL-2 cytokine. Here, we have shown that there is another activator of such lymphocytes, which is released by the immune system cells, namely, the innate immunity protein, Tag7. Perhaps, there are other regulators of cytotoxic CD4+CD25+ lymphocytes, and their identification is essential to understand the mechanisms of immune defense.

It is interesting whether there is a connection between the cytotoxic CD4+CD25+ cells we described in the Tag7-activated population and the regulatory CD4+CD25+ lymphocytes (Treg). As was mentioned in Introduction, Treg blocks the activity of the effector T lymphocytes not only through the secretion of suppressor cytokines. They can also directly kill lymphocytes by secretion of granzymes. We investigated whether Treg can kill tumor cells via the FasL-Fas interaction. We showed that the CD4+CD25+CD127− cell fraction did not demonstrate cytotoxic activity. Tumor cells were killed only by the Cd4+CD25+CD127+ cells. Therefore, if Treg can kill cells directly, they kill only the effector T lymphocytes. They do not kill HLA-positive tumor cells, although they could affect their level in the body by affecting the effector T lymphocytes.

## Supplementary Materials

Supplementary Materials Supplemental Figure 1: cytolytic activity increases with purification. The CD4+ population was isolated from the 6-day LAK culture with antibody-coated magnetic beads, and the CD4+CD25+ subset was similarly isolated from CD4+. Both were incubated with K562 in standard assays at different effector-target cell ratios. Supplemental Figure 2: gating strategy for isolating the lymphocyte population from PBMC cells for Figure 1. A. Lymphocyte gating. B. CD3CD4 gating. E2-double-positive cells. C. CD3CD8 gating. E2-double-positive cells. Supplemental Figure 3: gating strategy for isolating the lymphocyte population from CD4+CD25+ T lymphocyte subpopulation, purified on magnetic beads, for Figure 3. A. Lymphocyte gating. B. Staining by the FITC-conjugated anti-rabbit antibodies. Supplemental Figure 4: gating strategy for isolating the lymphocyte population from PBMC cells. Supplemental Figure 5: the CD16+CD56+ population was isolated from the 4-day LAK culture with antibody-coated magnetic beads. Cells were incubated with K562 in standard assays with 1-hour preincubation with antibodies to FasL, Fas, and granzyme B. Supplemental Figure 6: the CD3+CD8+ population was isolated from the 4-day LAK culture with antibody-coated magnetic beads. Cells were incubated with K562 in standard assays with 1-hour preincubation with antibodies to FasL, Fas, granzyme B, Tag7, and Hsp70. Supplemental Figure 7: flow cytometry of FoxP3 intracellularly stained with the mouse anti-FoxP3 antibodies followed by the PE-conjugated anti-mouse antibodies. A. The CD4+CD25+CD127+ population stained by PE-conjugated anti-mouse antibodies. B. The CD4+CD25+CD127+ population stained with the mouse anti-FoxP3 antibodies followed by PE-conjugated anti-mouse antibodies. C. Total PBMC population stained with the mouse anti-FoxP3 antibodies followed by PE-conjugated anti-mouse antibodies. Supplemental Figure 8: gating strategy for isolating the Tag7+ lymphocyte population from PBMC, purified on magnetic beads. A. Lymphocyte gating. B. Staining with the mouse anti-granzyme B antibodies followed by PE-conjugated anti-mouse antibodies.

## References

[1] Seliger B. (2005). Strategies of tumor immune evasion. *BioDrugs*.

[2] Bubeník J. (2004). MHC class I down-regulation: tumour escape from immune surveillance? (review). *International Journal of Oncology*.

[3] Markel G., Lieberman N., Katz G. (2002). CD66a interactions between human melanoma and NK cells: a novel class I MHC-independent inhibitory mechanism of cytotoxicity. *The Journal of Immunology*.

[4] Brutkiewicz R. R. (2006). CD1d ligands: the good, the bad, and the ugly. *The Journal of Immunology*.

[5] Yu K. O. A., Porcelli S. A. (2005). The diverse functions of CD1d-restricted NKT cells and their potential for immunotherapy. *Immunology Letters*.

[6] Kunzmann V., Wilhelm M. (2005). Anti-lymphoma effect of *γδ* T cells. *Leukemia & Lymphoma*.

[7] Meresse B., Chen Z., Ciszewski C. (2004). Coordinated induction by IL15 of a TCR-independent NKG2D signaling pathway converts CTL into lymphokine-activated killer cells in celiac disease. *Immunity*.

[8] Nausch N., Cerwenka A. (2008). NKG2D ligands in tumor immunity. *Oncogene*.

[9] Talebian L., Fischer D. A., Wu J. (2014). The natural killer–activating receptor, NKG2D, on CD3+CD8+ T cells plays a critical role in identifying and killing autologous myeloma cells. *Transfusion*.

[10] Sashchenko L. P., Dukhanina E. A., Shatalov Y. V. (2007). Cytotoxic T lymphocytes carrying a pattern recognition protein Tag7 can detect evasive, HLA-negative but Hsp70-exposing tumor cells, thereby ensuring FasL/Fas-mediated contact killing. *Blood*.

[11] Appay V., Zaunders J. J., Papagno L. (2002). Characterization of CD4^+^ CTLs ex vivo. *The Journal of Immunology*.

[12] Laouar Y., Crispe I. N. (2000). Functional flexibility in T cells: independent regulation of CD4^+^ T cell proliferation and effector function in vivo. *Immunity*.

[13] Takeuchi A., Saito T. (2017). CD4 CTL, a cytotoxic subset of CD4^+^ T cells, their differentiation and function. *Frontiers in Immunology*.

[14] Walker M. R., Kasprowicz D. J., Gersuk V. H. (2003). Induction of FoxP3 and acquisition of T regulatory activity by stimulated human CD4^+^CD25^–^ T cells. *The Journal of Clinical Investigation*.

[15] von Boehmer H. (2005). Mechanisms of suppression by suppressor T cells. *Nature Immunology*.

[16] Larin S. S., Korobko E. V., Kustikova O. S. (2004). Immunotherapy with autologous tumor cells engineered to secrete Tag7/PGRP, an innate immunity recognition molecule. *The Journal of Gene Medicine*.

[17] Dziarski R. (2004). Peptidoglycan recognition proteins (PGRPs). *Molecular Immunology*.

[18] Kustikova O. S., Kiselev S. L., Borodulina O. R., Senin V. M., Afanas'eva A. V., Kabishev A. A. (1996). Cloning of the tag7 gene expressed in metastatic mouse tumors. *Genetika*.

[19] Kang D., Liu G., Lundstrom A., Gelius E., Steiner H. (1998). A peptidoglycan recognition protein in innate immunity conserved from insects to humans. *Proceedings of the National Academy of Sciences of the United States of America*.

[20] Michel T., Reichhart J. M., Hoffmann J. A., Royet J. (2001). *Drosophila* toll is activated by gram-positive bacteria through a circulating peptidoglycan recognition protein. *Nature*.

[21] Sashchenko L. P., Dukhanina E. A., Yashin D. V. (2004). Peptidoglycan recognition protein tag7 forms a cytotoxic complex with heat shock protein 70 in solution and in lymphocytes. *The Journal of Biological Chemistry*.

[22] Dukhanina E. A., Lukyanova T. I., Romanova E. A. (2015). A new role for PGRP-S (Tag7) in immune defense: lymphocyte migration is induced by a chemoattractant complex of Tag7 with Mts1. *Cell Cycle*.

[23] Sharapova T. N., Ivanova O. K., Soshnikova N. V., Romanova E. A., Sashchenko L. P., Yashin D. V. (2017). Innate immunity protein Tag7 induces 3 distinct populations of cytotoxic cells that use different mechanisms to exhibit their antitumor activity on human leukocyte antigen-deficient cancer cells. *Journal of Innate Immunity*.

[24] Sashchenko L. P., Gnuchev N. V., Lukjanova T. I. (1993). Time-dependent changes of LAK cell phenotypes correlate with the secretion of different cytotoxic proteins. *Immunology Letters*.

[25] Sashchenko L. P., Romanova E. A., Ivanova O. K., Sharapova T. N., Yashin D. V. (2017). FasL and the NKG2D receptor are required for the secretion of the Tag7/PGRP-S–Hsp70 complex by the cytotoxic CD8^+^ lymphocytes. *IUBMB Life*.

[26] Sakaguchi S. (2005). Naturally arising Foxp3-expressing CD25^+^CD4^+^ regulatory T cells in immunological tolerance to self and non-self. *Nature Immunology*.

[27] Liu W., Putnam A. L., Xu-yu Z. (2006). CD127 expression inversely correlates with FoxP3 and suppressive function of human CD4^+^ T reg cells. *Journal of Experimental Medicine*.

[28] Wagner H., Starzinski-Powitz A., Jung H., Röllinghoff M. (1977). Induction of I region-restricted hapten-specific cytotoxic T lymphocytes. *The Journal of Immunology*.

[29] Feighery C., Stastny P. (1979). HLA-D region-associated determinants serve as targets for human cell-mediated lysis. *Journal of Experimental Medicine*.

[30] Zaunders J. J., Dyer W. B., Wang B. (2004). Identification of circulating antigen-specific CD4^+^ T lymphocytes with a CCR5^+^, cytotoxic phenotype in an HIV-1 long-term nonprogressor and in CMV infection. *Blood*.

[31] Aslan N., Yurdaydin C., Wiegand J. (2006). Cytotoxic CD4^+^ T cells in viral hepatitis. *Journal of Viral Hepatitis*.

[32] Brown D. M., Lee S., Garcia-Hernandez M. L., Swain S. L. (2012). Multifunctional CD4 cells expressing gamma interferon and perforin mediate protection against lethal influenza virus infection. *Journal of Virology*.

[33] Xie Y., Akpinarli A., Maris C. (2010). Naive tumor-specific CD4^+^ T cells differentiated in vivo eradicate established melanoma. *Journal of Experimental Medicine*.

[34] van de Berg P. J., van Leeuwen E. M., ten Berge I. J., van Lier R. (2008). Cytotoxic human CD4^+^ T cells. *Current Opinion in Immunology*.

[35] Thewissen M., Somers V., Hellings N., Fraussen J., Damoiseaux J., Stinissen P. (2007). CD4^+^CD28^null^ T cells in autoimmune disease: pathogenic features and decreased susceptibility to immunoregulation. *The Journal of Immunology*.

[36] Quezada S. A., Simpson T. R., Peggs K. S. (2010). Tumor-reactive CD4^+^ T cells develop cytotoxic activity and eradicate large established melanoma after transfer into lymphopenic hosts. *Journal of Experimental Medicine*.

[37] González S., López-Soto A., Suarez-Alvarez B., López-Vázquez A., López-Larrea C. (2008). NKG2D ligands: key targets of the immune response. *Trends in Immunology*.

[38] Ashiru O., Boutet P., Fernández-Messina L. (2010). Natural killer cell cytotoxicity is suppressed by exposure to the human NKG2D ligand MICA^∗^008 that is shed by tumor cells in exosomes. *Cancer Research*.

[39] Hantschel M., Pfister K., Jordan A. (2000). Hsp70 plasma membrane expression on primary tumor biopsy material and bone marrow of leukemic patients. *Cell Stress & Chaperones*.

[40] Ferrarini M., Heltai S., Zocchi M. R., Rugarli C. (1992). Unusual expression and localization of heat-shock proteins in human tumor cells. *International Journal of Cancer*.

[41] Gehrmann M., Liebisch G., Schmitz G. (2008). Tumor-specific Hsp70 plasma membrane localization is enabled by the glycosphingolipid Gb3. *PLoS One*.

[42] Beal A. M., Anikeeva N., Varma R. (2009). Kinetics of early T cell receptor signaling regulate the pathway of lytic granule delivery to the secretory domain. *Immunity*.

