# Supplementary material for: Tag7 (PGLYRP1) Can Induce an Emergence of the CD3+CD4+CD25+CD127+ Cells with Antitumor Activity
Source: J Immunol Res. 2018 Apr 11;2018:4501273. doi: 10.1155/2018/4501273 (PMC5925135; doi:10.1155/2018/4501273)
Supplement: Supplementary Materials — Supplemental Figure 1: cytolytic activity increases with purification. The CD4+ population was isolated from the 6-day LAK culture with antibody-coated magnetic beads, and the CD4+CD25+ subset was similarly isolated from CD4+. Both were incubated with K562 in standard assays at different effector-target cell ratios. Supplemental Figure 2: gating strategy for isolating the lymphocyte population from PBMC cells for Figure 1. A. Lymphocyte gating. B. CD3CD4 gating. E2-double-positive cells. C. CD3CD8 gating. E2-double-positive cells. Supplemental Figure 3: gating strategy for isolating the lymphocyte population from CD4+CD25+ T lymphocyte subpopulation, purified on magnetic beads, for Figure 3. A. Lymphocyte gating. B. Staining by the FITC-conjugated anti-rabbit antibodies. Supplemental Figure 4: gating strategy for isolating the lymphocyte population from PBMC cells. Supplemental Figure 5: the CD16+CD56+ population was isolated from the 4-day LAK culture with antibody-coated magnetic beads. Cells were incubated with K562 in standard assays with 1-hour preincubation with antibodies to FasL, Fas, and granzyme B. Supplemental Figure 6: the CD3+CD8+ population was isolated from the 4-day LAK culture with antibody-coated magnetic beads. Cells were incubated with K562 in standard assays with 1-hour preincubation with antibodies to FasL, Fas, granzyme B, Tag7, and Hsp70. Supplemental Figure 7: flow cytometry of FoxP3 intracellularly stained with the mouse anti-FoxP3 antibodies followed by the PE-conjugated anti-mouse antibodies. A. The CD4+CD25+CD127+ population stained by PE-conjugated anti-mouse antibodies. B. The CD4+CD25+CD127+ population stained with the mouse anti-FoxP3 antibodies followed by PE-conjugated anti-mouse antibodies. C. Total PBMC population stained with the mouse anti-FoxP3 antibodies followed by PE-conjugated anti-mouse antibodies. Supplemental Figure 8: gating strategy for isolating the Tag7+ lymphocyte population from PBMC, purified on magnetic beads. A [file 4501273.f1.docx]

Supplemental Figures.


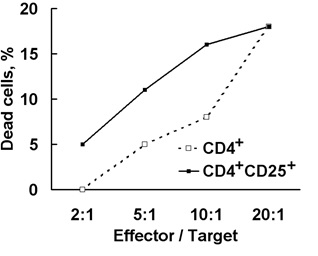


**Supplemental figure 1.**

Cytolytic activity increases with purification. The CD4+ population was isolated from the 6-day LAK culture with antibody-coated magnetic beads, and the CD4+CD25+subset was similarly isolated from CD4+. Both were incubated with K562 in standard assays at different effector–target cell ratios.


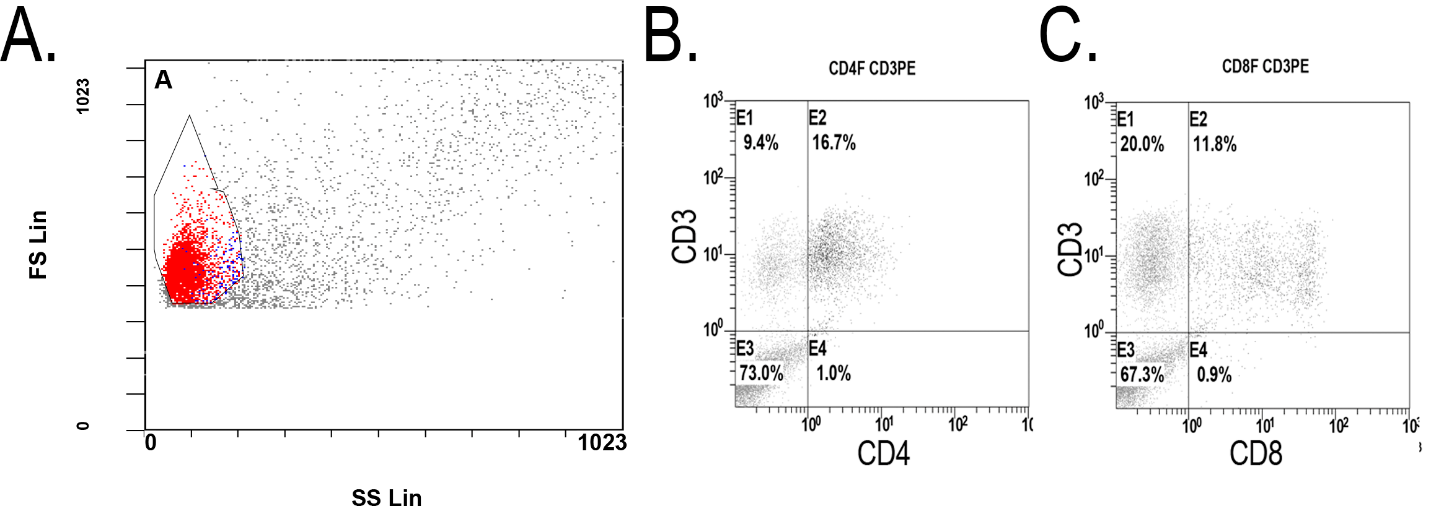


**Supplemental figure 2.**

Gating strategy for isolating the lymphocytes population from PBMC cells for Figure 1. A. Lymphocytes gating. B. CD3CD4 gating. E2 – double positive cells. C. CD3CD8 gating. E2-double positive cells.

**
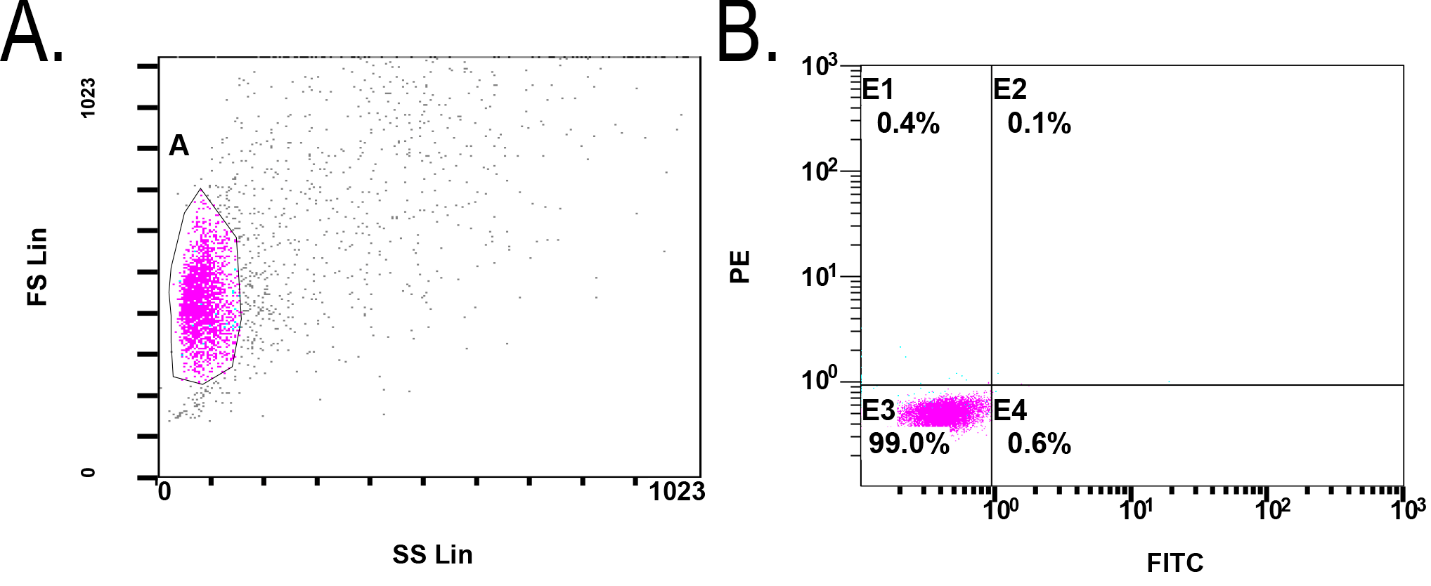
**

**Supplemental figure 3.**

Gating strategy for isolating the lymphocytes population from CD4+CD25+ T lymphocytes subpopulation, purified on magnetic beads, for Figure 3. A. Lymphocytes gating. B. Staining by the FITC-conjugated anti-rabbit antibodies.


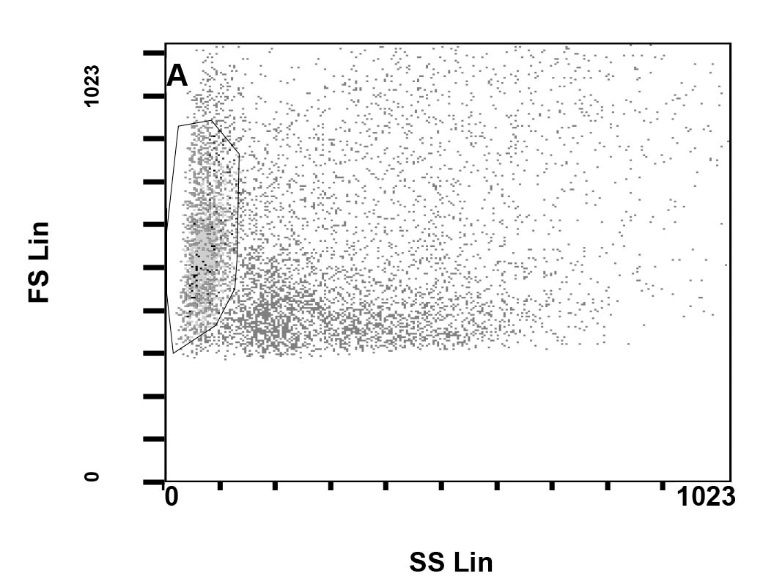


**Supplemental figure 4.**

Gating strategy for isolating the lymphocytes population from PBMC cells.





**Supplemental figure 5.**

The CD16+CD56+ population was isolated from the 4-day LAK culture with antibody-coated magnetic beads. Cells were incubated with K562 in standard assays with 1hour preincubation with antibodies to FasL, Fas, Granzyme B.





**Supplemental figure 6.**

The CD3+CD8+ population was isolated from the 4-day LAK culture with antibody-coated magnetic beads. Cells were incubated with K562 in standard assays with 1hour preincubation with antibodies to FasL, Fas, Granzyme B, Tag7 and Hsp70.


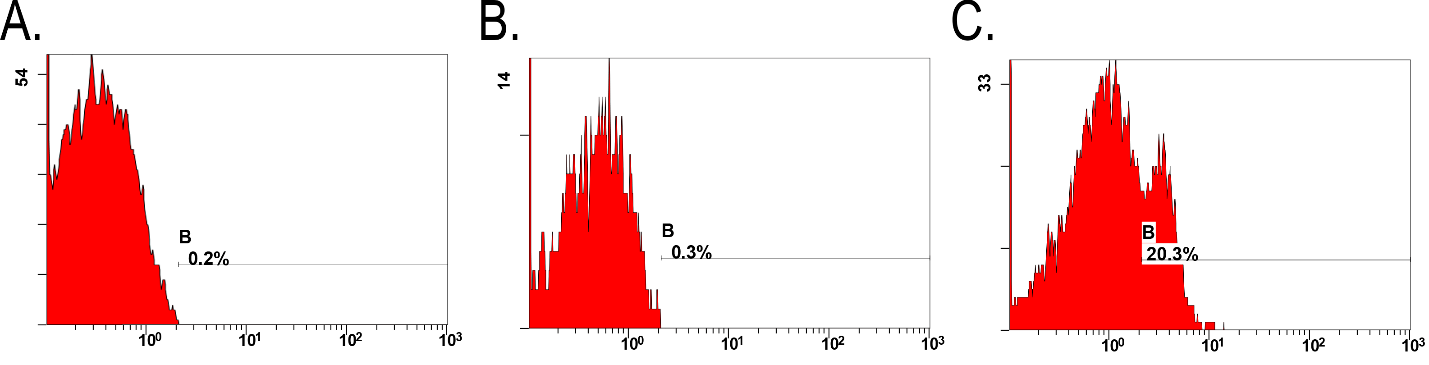


**Supplemental figure 7.**

Flow cytometry of FoxP3 intracellulary stained with the mouse anti-FoxP3 antibodies following by the PE-conjugated anti-mouse antibodies. A. the CD4+CD25+CD127+ population stained by PE-conjugated anti-mouse antibodies. B. the CD4+CD25+CD127+ population stained with the mouse anti-FoxP3 antibodies following by PE-conjugated anti-mouse antibodies. C. total PBMC population stained with the mouse anti-FoxP3 antibodies following by PE-conjugated anti-mouse antibodies.





**Supplemental figure 8.**

Gating strategy for isolating the Tag7+ lymphocytes population from PBMC, purified on magnetic beads. A. Lymphocytes gating. B. Staining with the mouse anti-Granzyme B antibodies following by PE-conjugated anti-mouse antibodies.
